# No ordinary adult life: Living conditions from the perspective of adults with intellectual disabilities

**DOI:** 10.1177/17446295221107284

**Published:** 2022-06-11

**Authors:** Õie Umb Carlsson, Päivi Adolfsson

**Affiliations:** Department of Public Health and Caring Sciences, Disability and Habilitation, 8097 Uppsala University, Sweden; Department of Public Health and Caring Sciences, Health Equity and Working Life/HEAL, 8097 Uppsala University, Sweden; Department of Public Health and Caring Sciences, Centre for Disability Research, 8097 Uppsala University, Sweden

**Keywords:** intellectual disabilities, living conditions, quality of life, self-report

## Abstract

This study focuses on the subjective experience of the living conditions of adults with intellectual disabilities as related to the Uppsala Quality of Life model (UQoL2). Eleven semi-structured interviews were conducted to study issues raised by people with intellectual disabilities. Study participants had their own home, either in an ordinary dwelling or group home. The findings show that the dependence on support in daily life infringes on the sense of adult social status and control of life. Staff and family had a mandate to define Quality of Life, which countered the possibilities of a life based on the preferences of those with intellectual disabilities. Knowledge about factors that affect living conditions, one of the social determinants of health, has implications for public health in general and the development of society’s efforts for people who are in lifelong dependence on support and service from others.

## Introduction

Living conditions in the Swedish adult population are constantly changing over time. This constant change also applies to people with intellectual disabilities. Today, because all larger segregated institutions and nursing homes are closed, people with intellectual disabilities live in the community where other members of society live (Statistics Sweden [SCB], 2021). They are eligible to apply for services and support in housing, employment and leisure. The Swedish Act Concerning Support and Service for Persons with Certain Functional Impairments (SFS 1993:387) and disability policy (Ministry of Health and Social Affairs, 2017) are underpinned by the principle of equal value of all people and recognize the opportunity for equal living conditions for people with and without disabilities.

Studies, both in Sweden and internationally, have compared living conditions of people with and without disabilities (Barranha et al.,2021; Emerson et al., 2012; Pérez-Hernández et al., 2019; Umb Carlsson and Sonnander, 2005). Some studies focus on objective living conditions in various domains: housing, occupation, education, finances, leisure, personal safety and social belonging (Umb Carlsson, 2020; Umb-Carlsson and Sonnander, 2005). Other studies pay attention to peoples’ experiences of their everyday lives (cf. Witsø and Hauger, 2020) and what contributes to a quality life (Umb Carlsson and Adolfsson, 2018).

Empirical findings indicate that people with disabilities are disadvantaged in various domains, with fewer having a paid employment (Giri et al., 2021; Umb-Carlsson and Sonnander, 2005), more having poor finances (Conder and Mirfin-Veitch, 2020; Umb-Carlsson and Sonnander, 2005; Williams and Porter, 2017) and more having a limited social life (Scott et al., 2014; Umb-Carlsson and Sonnander, 2005). Many people with intellectual disabilities depend on others in daily life. Being reliant on others may reduce a person’s possibilities of being treated as an individual with needs, desires and ambitions (O’Donovan et al., 2017). Living in a group home apartment may demand rationalizations and stereotypical solutions, often diminishing the role of the individual (Adolfsson et al., 2012). Thus, support from others (e.g. family members, housing staff) may have an obstructive impact on control of life (Björnsdóttir et al., 2015) and community participation (Simplican et al., 2015; Williams and Poerter, 2017).

A review of the literature suggests that many measures and ratings on the quality of life (QoL) and living conditions of individuals with intellectual disabilities focus the perspective of proxy’s (Sheridan et al., 2020). However, some evidence show differences between proxy reports and self-reports indicating that these are not equivalent and may capture different perspectives (Claes et al., 2012). The reliability of proxy ratings are questionable especially when it comes to subjective measures such as personal experiences of living conditions and QoL (Romhild et al., 2018). The views of people with intellectual disabilities are essential for understanding their experiences, needs and preferences.

Empirical findings indicate that measures taking the starting point in the perspective of people with intellectual disabilities may identify factors that have not otherwise been noticed (Umb Carlsson and Adolfsson, 2018). UQoL2 is a model based on data from people with intellectual disabilities in individual interviews and focus group discussions (Umb Carlsson and Adolfsson, 2018). The model includes components that contribute to wellbeing, of which control of life, social belonging and personal safety are equivalent to those frequently emphasized in the literature. The fourth component, - adult social status - is rarely explicitly addressed in other QoL measures and highlights the importance of including people with intellectual disabilities’ lived experiences when designing measures (Umb Carlsson and Adolfsson, 2018).

In previous studies the living conditions of a selected group of people with intellectual disabilities were reported from a proxy view (Umb Carlsson, 2020; Umb-Carlsson and Sonnander, 2005). In this study, we focus on experienced living conditions from the perspective of adults with intellectual disabilities. This study adds to the evidence base because previous studies have not researched the views of people with intellectual disabilities directly. There is a risk previous studies have missed important things because they have only asked proxies.

## Method

A qualitative approach was used with semi-structured interviews to collect information on the living conditions of persons with intellectual disabilities. In order to address issues relevant for the target group, the interview guide and the analyses model was developed by people with intellectual disabilities (cf Tøssebro, 1998).

### Participants

A purposive sample of adults with intellectual disabilities was recruited from participants in a 16-year follow-up study on living conditions (Umb Carlsson, 2020). To obtain a variety of perspectives, participants with different sex, age and life situations were invited (Gates and Waight, 2007). The only inclusion criterion was to be able to communicate with the interviewer. An information letter was sent to 14 persons and their parents/trustees that included information about the study and a participation request. Of these 14 adults, 11 gave written informed consent in consultation with a parent/trustee. For two persons (one woman and one man), participation was declined because they could not understand and answer questions due to dementia. One man was excluded due to severe intellectual disabilities and limited communicative ability. All 11 study participants were provided staff support at home (Table 1). Study participants living in a group home had a private apartment and access to a common area.

**Table 1.** Sociodemographic characteristics of participants

Pseudonyme	Age	Housing	Need assistance when outside home	Level of functional limitations^1^	Contact with parent/-s	Contact with siblings	Trustee	Contact person or companion service at leisure	Transportation service
Anne	54	Group home^2^	When outside the domicile	Severe	Weekly	Rarely or never	Yes	Yes	Yes
Karen	51	Group home^2^	Always	Moderate	--^3^	Rarely or never	Yes	Yes	Yes
Lisa	45	Ordinary with personal assistant	Always	Severe	Weekly	Monthly	No	No	No
Rosie	46	Group home^2^	When outside the domicile	Moderate	Weekly	Rarely or never	Yes	No	Yes
Catrine	45	Group home^2^	Manage herself	Moderate	Weekly	Rarely or never	Yes	Yes	Yes
Louise	46	Ordinary with personal assistant	When outside the domicile	Mild	Weekly	Monthly	No	No	No
Carl	46	Ordinary with housing support	When outside the domicile	Mild	Weekly	Rarely or never	Yes	Yes	No
Mike	49	Group home^2^	When outside the domicile	Severe	Weekly	Monthly	Yes	No	No
Jan	54	Ordinary with personal assistant	Always	Severe	Weekly	Monthly	Yes	No	No^4^
Tom	59	Ordinary with housing support	When outside the domicile	Mild	Weekly	Monthly	Yes	Yes	Yes
Paul	59	Group home^2^	Always	Moderate	Weekly	Rarely or never	Yes	Yes	Yes

^1^Assessed by relatives and staff who were well acquainted with the living conditions of the person with intellectual disabilities

^2^A private apartment with access to a common area

^3^No parent alive

^4^Has a car but no driving license

The study was approved by the Regional Ethical Review Board in Uppsala County (Reg. no.2016/319)

### Procedure

The first author conducted all interviews at a place and time chosen in agreement with the study participants. Eight interviews were carried out in the study participants’ homes, two in the workplaces and one by phone: the interviews, which took 1-2 hours, were conducted in May-June 2019.

Each interview began by repeating the information about the study and a reminder that their participation was voluntary. To encourage study participants to express their perspectives in their own words and avoid complimentary remarks from a significant other, the ambition was that the interviews would be conducted without the participation of a third person. However, at the request of three study participants, a trustee participated as support to help the participant to understand the questions. The trustees were not involved in providing information on behalf of the study participant. During one interview, however, a housing staff participated at the end to provide demographic information.

Interviews were directed by an interview guide containing 65 questions (Appendix 1) on housing, occupation, finances, leisure activities and family and social relations. The guide was compiled based on a questionnaire used in Umb Carlsson (2020). The questionnaire was developed by two groups of adults with intellectual disabilities to obtain information from proxies on the living conditions of adults with intellectual disabilities. Detailed information on the procedure and the questionnaire has been reported elsewhere (Umb Carlsson, 2020). In order to obtain face validity, the two groups of adults with intellectual disabilities who developed the questionnaire also compiled the interview guide for the present study. They formulated a detailed interview guide with issues that they considered relevant for the experiences of living conditions (Appendix 1). The ambition was to formulate questions that as many people with intellectual disabilities as possible could answer but it was emphasized that the wording had to be adjusted to each interviewee with relevant follow-up questions and probes. The preliminary version of the interview guide was discussed in eight meetings by five adults with intellectual disabilities in a municipality in eastern Sweden. Based on these discussions, minor changes in wording were made to align it with the aim of the present study.

Interview questions were open-ended. Study participants were free to discuss other issues related to living conditions whenever they wanted. Interviews were conducted flexibly considering the communicative ability of each study participant. Photos and personal attributes in the private home were used to facilitate communication. All interviews were recorded and transcribed verbatim by the first author.

### Analysis

**Figure 1.**
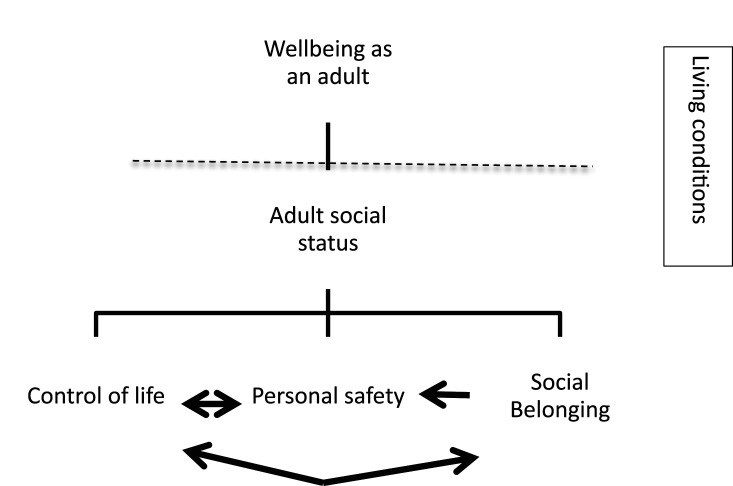
The UQoL2 model (Umb Carlsson and Adolfsson, 2018).

The UQoL2 model was chosen as an appropriate base to code the experiences of living conditions (Figure 1). The model evolves through the whole coding process. The analyses go beyond the verbally communicated data to identify the features of QoL. The aim was to identify information relevant to each component.

The coding process was collaborative, with both authors involved in the analytic process (Braun and Clarke, 2021). Information that could identify study participants was anonymized. Names and places that could identify study participants were removed. Both authors read the interviews repeatedly to secure a good overview of the material. To determine study participants' perspectives regarding their living conditions the material was analyzed in two steps by the second author using deductive top down thematic analysis (Braun and Clarke, 2006) in NVIVO software. In the first step, the material was sorted in clusters according to the UQoL2: wellbeing, adult social status, control of life, personal safety and social belonging. The result of this sorting was discussed by the authors until consensus was reached. As a second step, the material from the first sorting was sorted once more. This time the material from each UQoL2 cluster was sorted in the areas included in the interview guide (Appendix 1). After this process, the first author reviewed the material within each group separately. The authors discussed the content, and the interviews were reread to ensure the validity of the analysis.

## Results

Results are presented based on the UQoL2 model (Umb Carlsson and Adolfsson, 2018). The UQoL2 is made up of four components: adult social status, control of life, personal safety and social belonging. The components apply to the subjective experiences of living conditions in several life areas: housing, occupation, finances, leisure activities, and family and social relations. In the following we report issues designated as essential within each UQoL2 component. Quotations are used to illustrate and exemplify the findings. Study participants’ names are replaced with pseudonyms. In some cases the interviewer’s question has been included to clarify the information. This scheme is noted by the letter I.

Reported quotes are distributed unevenly between study participants, reflecting their ability to express themselves verbally rather than the amount of information they have contributed.

### Adult social status

In the UQoL2 the component adult social status is underscored as the main constituent of wellbeing (Umb Carlsson and Adolfsson, 2018). Experiencing adult social status comprises control of life, personal safety and social belonging and, more importantly, being respected for who you are as a person, with shortcomings and strengths. A prerequisite is to have access to objective living conditions typical for adults, such as a home of one’s own, a paid job and the opportunity to practice age-adequate recreational activities (Umb Carlsson and Adolfsson, 2018).

In the present study participants emphasized the importance of others trusting their ability to take responsibility and make independent and critical judgments. For example, this was illustrated by having the opportunity to shape the private apartment according to personal preferences. Another example was to have the chance to co-habit with a partner or a friend, which they had chosen, no matter what others thought about their choice. Louise was dissatisfied that her mother and mother-in-law were interfering in the way she and her co-habitant lives.

Louise: My mother and his mother, so my mother-in-law, they quarrel the most… Edward and I are, we are, mean is sad, pissed because they are nagging us, Edward and me. We cannot stand it, it’s their fault. Edward and I are happy… are in love.

Study participants experienced facilitators and barriers in their quest to seize the opportunity to enter into a responsible adult role and choose an acceptable lifestyle. The housing staff had set the framework but, at the same time, encouraged study participants to act within these regulations. Although staff had chosen which day of the week daily chores in the private home should be performed, study participants experienced adult status by staff showing confidence in their ability to perform everyday chores. Tom was happy to be considered able to clean his apartment himself: *Sometimes I have already cleaned when they arrive (Tom*). On the other hand, staff showed a lack of confidence that the participants could decide themselves whether it was appropriate to go out in the evening or not. *I: If you want to go out in the evening, do you have to ask the staff then? Karen: Yes, it is.*

The staff decided how the common area of the group home was to be used. If the staff had a meeting or needed to exchange information, study participants could not access this part of their home. Thus, “the home” did not include the whole dwelling in which the individual lived. Instead, it was limited to “the private apartment”, regardless of whether it was located within the common area or close to it. This illustrates a dilemma in that some activities usually associated with one’s private home, such as everyday meals and watching TV in the evenings occured outside “the home”.

At work, study participants had the opportunity to influence tasks, work pace and other work-related circumstances. This possibility contributed to a sense of having adult responsibility and that others felt confident in their ability to decide what worked best. Louise exemplified by her experience of being listened to when her working hours did not work well.

Louise: So, I was with work nine to two …It’s too early for me…Yes, too early. Now I have, I’ve talked to supervisor… I’ve talked to John, it has worked very well this year… Now come to work at eleven…Yes like this, the problem is last year. I was, yes came late every day, every month…I tried ten times but late it was… I got tired and stressed…This year much better. Last year no.

Moreover, study participants felt they were given adult responsibility to decide about sick leave and inform the workplace and housing staff.

Tom: Yes, then you can be at home. Then I call to the work and to the housing staff. Tell them that I am at home.

Experience of adult social status is linked to making decisions and acting for oneself and benefiting others. Some study participants told that family members provided help when needed but also the reverse, they asked for help when needed. For example, Cathrine, was entrusted to take care of the parent’s dogs and house while the parents were engaged in other activities: *I usually help with babysitting, dog sitting…At mum. They have two dogs.* This trust in her ability to provide help contributed to a sense of being included in the adult social community by her family.

### Control of life

According to UQoL2 (Umb Carlsson and Adolfsson, 2018), control of life means being the ‘master of everyday life’ and to live a life according to one’s values and preferences. Participation and involvement are prerequisites in decisions such as where to live and work. To have control of life does not necessarily mean acting independently; rather, it entails influencing whether one can manage without help (Umb Carlsson and Adolfsson, 2018).

Study participants expressed a desire to control their life course and opposed others taking control over their lifestyle. However, they illustrated control of life differently, giving it meaning at diverse levels. For example, study participants living in a group home put control of life in relation to everyday matters. They talked about having influence over the weekly menu and choice over where and with whom to dine. Anne lived in a group home. She did not feel comfortable with her co-residents and usually chose not to spend time with them. *I: Do you have coffee with the others living in the group-home? Anne: No, I don’t want to.* Study participants living in ordinary housing, on the other hand, related control of life to general issues, such as service provision and the opportunity to replace a staff member if they were dissatisfied with that person.

I: If there is an assistant you don’t like, what do you do then?

Jan: Then you try to talk to her what she’s doing wrong…starting with… and if it does not work, you have to change then.

Louise, living in ordinary housing with support from personal assistants, said that she and her co-habitant valued different characteristics of the housing staff. They therefor employed different assistant companies, which gave both her and her co-habitant control over their personal support. *His assistance is [company name]and my assistance helps me… It works well. (Louise)*

At work, control was related to influencing the planning of a working day. According to the study participants, a written plan over the work situation contributed to predictability and increased their experience of control over working life. A prerequisite, however, was having a responsive staff that showed care and listened to the individual’s wishes and preferences. Study participants using verbal communication found it easier to have their wishes met (exemplified with the quote with Anne) compared to those with limited verbal communication (Lisa).

I: Is it fun at work?

Anne: No, I do not want stay…I have to, I have to change… I will package, sort the letters. That’s what to do.

I: Would it be fun?

Anne: Yes of course.

Lisa rarely expressed her own desires verbally. She was dependent on staff and family listening to her body-language and their aspiration to see when she was not happy.

I: Would you like to change job?

Lisa: (Nods) I want to keep painting.

I: Can you do it where you are now?

Lisa: No.

I: Have you told the staff that you want to change job?

Lisa: No

I: Do you think you would get it, if you told?

Lisa: (Nods)

In the UQoL2 people with intellectual disabilities emphasized that being dependent on others may inhibit choice of lifestyle, social interaction and participation in society (Umb Carlsson and Adolfsson, 2018). In the present study participants specified that they were not forced to join activities that were not of value or interest. Despite this, the study participants experienced limited control over leisure activities. The need for support restricted their opportunities for activities (e.g., visiting concerts, dancing, strolling in town). Receiving support from staff or contact persons was not always available. Cathrine lived in a group home:

Cathrine: If it is light on day then I can go out for a walk. Not late in the evening…Mom does not allow.

I: But if you want to go out in the evening, is there anyone who can accompany you then?

Cathrine: No, none

I: No, so you cannot go out in the evening when it is dark?

Cathrine: No

### Personal safety

According to the UQoL2 (Umb Carlsson and Adolfsson, 2018), personal safety is conditional on adult social status, control of life and social belonging. Services and support should be individually tailored and last over time. They should be governed by the needs of the individual at different time points and not by changes in political-economic attitudes (Umb Carlsson and Adolfsson, 2018).

The interviews indicate that the experience of safety depended on how services and support were provided and the staff attitude. Factors contributing to the experience of personal safety were 1) predictability, 2) influence on how and when everyday chores were performed and 3) continuity in staff support. In the private apartment, a weekly schedule of leisure activities and staffing contributed to predictability. At daily activities, knowledge of routines and rules helped to make workday more predictable.

Overall, study participants expressed their trust in the staff’s ability to know everyday matters and provide adequate support. One exception, however, was that the staff were not given authority to handle the study participants’ money. Parents and trustees ensured finances and that the money was enough for both necessary and extra expenses. Rosie’s aunt was her trustee and she was confident that her aunt would ensure that the money was enough for necessary expenses.

Rosie: I ask my aunt if I can take out more money. I go to the ATM and take out. And she said, it is okay to take out five hundred. If not, if not I may not take out more.

Although study participants felt trust in staff having knowledge on adequate everyday issues such as washing and cleaning, they wanted to have control over health-promoting measures. Some had acquired knowledge which enabled own responsibility for health and wellbeing. Cathrine, for example, had knowledge about which foods she was allergic to. This knowledge reduced her uncertainty and vulnerability it otherwise would have implied to be utterly dependent on staff members.

I: Do you eat with the others living in the group home?

Cathrine: No, not me but the others do… I have my own food.

I: You want your own food?

Cathrine: Yes, I am sensitized to milk and fat. Then I get really sick.

## Jan had severe physical problems following an accident:

I: Three days a week you’ll go to the habilitation and train?

Jan: Yes. That’s why I have become as good as I have become because of that then.

The UQoL2 emphasizes that a safe and secure home is chosen by the individuals and based on their preferences (Umb Carlsson and Adolfsson, 2018). This notion of safety in the home was also underlined in the present study. Study participants expressed that the private apartment was a place they usually found safe and secure. However, their experiences regarding safety were not unequivocal. They talked about insecurity in housing and at work due to the boisterous and disorderly behaviour of the co-residents/co-workers (e.g., fighting, insulting, screaming). Anne lived in a group home. Due to the behaviour of a co-resident, she had applied to move to another group home.

Anne: There is a lot of fuss and noise and…it’s just John got screaming too, it therefor hurt in the head…I do not want to stay here.

Although study participants noted that a sense of safety depended on their relationship to staff members and co-residents/co-workers, the fundamental factor regarding safety could be found in the family community. According to study participants, relationship in which individuals and their parents and siblings wanted to maintain social contact was crucial. Paul lived in a group home. His mother had visited him regularly throughout the years. When the mother could not continue with the visits for health reasons, a sibling took over. A brother made sure to maintain the relationship between Paul and his family. Thus, Paul was included in the family community.

Paul: But mother could not come at all, come back now instead because she has pain in her leg so she cannot move the leg and the muscle so she has tense it. She’s in a lot of pain, she cannot come it’s not possible… No, she’s not driving anymore… No, Alan [the brother] comes instead.

Rosie lived with her husband in a group home. They had been married for several years and Rosie felt safe to know that he was there for her.

I: Are you scared sometimes?

Rosie: Yes it, yes that too but not now anyway.

I: No, what are you afraid of then?

Rosie: If the man [her husband] is not here…If he is not still here.

I: Yes, you are afraid of that…He brings safety?

Rosie: I like him… I do.

### Social belonging

Social belonging involves interacting with other people, particularly with parents and siblings (Umb Carlsson and Adolfsson, 2018). According to UQoL2, a prerequisite for the success of social belonging is that relationships are reciprocal and valued by all parties.

Study participants had diverse experiences of being included in a family community. Their stories reflect close mutual relationships but also the absence of contact. Overall, others decided on the nature and frequency of contact, illustrating their dependency on the interests and goodwill of others. Cathrine wanted help in finding her biological mother. She raised concern about others deciding the suitability of arranging the contact with the birth mother.

Cathrine: Mom [foster mother] have finished thinking soon.

I: Okay, if she allows you to meet your biological mother, is that so?

Cathrine: Yes. But I want to meet her.

In contrast, study participants shared experiences that illustrated normal, mutual relationships with a family anxious to maintain contact.

Louise: I have a sister, too.

I: You have, yes. Do you usually meet her?

Louise: Yes, sometimes actually. Sometimes visit her.

I: Do you talk to her on the phone too?

Louise: Yes, yesterday.

In addition to relationships with family members, study participants indicated the importance of sustainability in contact with staff and friends. For example, Cathrine valued living in the same group home as her childhood friends.

Cathrine: They live here, too. Susan is her name Karlsson…She knows me, I was a small [child].

I: She knew you when you were a small child?

Cathrine: Yes, and Elisabeth know also, know since long.

I: You have known each other since long time?

Cathrine: Yes

Study participants living in a group home experienced that staff and residents contributed to social belonging. The staff promoted a sense of community by initiating and organizing shared meals and other joint activities in the dwelling’s common areas. Rosie appreciated that staff initiated various activities for those living in the group home: *And then coffee, or games or play games or movies or something like that… now it's read aloud… it's on Thursdays [in the common area]*.

In general, relationships outside the family were dependent on social support. For example, study participants addressed the need for support when being outside the home area. Thus, they experienced that activities outside the home were dependent on access to social support. In addition to staff in housing, contact persons provided social support, spending time and energy with the study participants. At the interviews, study participants described various activities they engaged in together with their contact person.

Anne: Yes, we meet in town, out for a coffee, then out to eat.

Mike: Going to the movies sometimes, watching football.

Karen: I’m going to sleep there, having coffee there [at the contact person’s home].

Paul: Dance.

However, if not appropriately implemented, social support could produce a feeling of exclusion. *Yes and then I have had another [contact person], her name is Susan. She has no time actually and see me* [Shows her disappointment non-verbally] (*Cathrine.)*

Of note, there were few stories about spending free time with selected co-workers and friends. Lisa had no friend who visited her in the home. *I do not often have anyone [visitor](Lisa).* Nevertheless, study participants emphasized that they wanted to choose those friends and others with whom to interact. They raised concern about contact solely based on similar disabilities and desired more activities contingent on shared interests with selected friends. During holidays, some study participants participated in special camps for people with intellectual disabilities with shared interests. They spoke of looking forward to meeting friends that they had not had contact with since the previous camp encounter.

For some study participants, being known in the local area contributed to a sense of social belonging in the community. Jan lived in as small municipality and was happy to be recognized when visiting the municipality centre. There was always someone who stopped and chatted for a while: *No I do not have much [friends] but I have a lot of people I know out here that I meet at the store (Jan).* For others, the alternative was to socialize via the Internet.

I: Your friend online. Have you ever met your friend?

Lisa: No.

I: No, only online?

Lisa: Yes.

I: Do you have another friend that you usually meet?

Lisa: No

### Wellbeing

In the UQoL2 model (Umb Carlsson and Adolfsson, 2018) wellbeing is considered a complex concept that includes good living conditions and satisfaction with their current standard of living. Attitudes, equality and tolerance may either decrease or increase an individual’s wellbeing. In addition, wellbeing includes the opportunity to live according to the individual’s preferences and values.

In the present study participants talked about wellbeing in terms of the totality of the life space. Sometimes things did not turn out well, leaving the study participants dissatisfied. It was important to be listened to and given the power to change the situation according to own preferences on such occasions.

Study participants emphasized that a key component of wellbeing was to be included in a social community with staff, other residents/co-workers and family. A friendship bond based on mutual interpersonal relations of trust and honesty increased a sense of social belonging. In contrast, they also told of being bullied and excluded from community activities and other events. They mentioned that such experiences impaired mental, social and physical wellbeing.

Louise: And that’s why I thought, my head, my whole body tired of me. I don’t want to hear that. Sad. I got angry. I’m pissed, my whole body feels and my back is shaky all the time on me. It feels uncomfortable for me.

Wellbeing involves the experience of satisfaction in different areas of life, including housing, daily activities and leisure time. Tom was happy that his application for changed tasks in daily activities was granted. *Tom: I*’*m glad I got that job, thrilled.* Despite enjoying the time at daily activities, study participants wanted to have a day off every week, i.e. a “home day*”. Cathrine: Yes, I work all days but not on Thursdays when I have a home day. I: Would you like to work on Thursdays as well? Cathrine: No.* During the home day, study participants were provided staff support to attend to domestic chores. More importantly, they were given an opportunity to participate in selected activities and own time with a staff member. Karen expressed great joy at having a coffee break alone with a staff: *Coffee here. Yes, coffee it, coffee, coffee, coffee it*’*s good [laughs and claps her hands of joy]*.

## Discussion

This qualitative study explored subjective experiences of living conditions from the perspective of middle-aged adults with intellectual disabilities in a Swedish county. Study participants described satisfaction with some areas of life such as housing and daily activities but expressed that they continue to feel excluded from the social community. They believed their individual preferences, autonomy and freedom were restricted by the need for support, which was not set up in way to maximize personalization. In accordance with previous findings (Fullana et al., 2020; Mallander, 2015), staff and family had a mandate to define what characterizes good living conditions and QoL, which countered the possibilities of a life based on own preferences. Thus, the dependence on support in daily life infringes on the sense of adult social status and control of life. Limited control over the life course and everyday matters has often been reported in the literature (Tait et al., 2020) and may emanate from a view of significant others having superior position of control. This corresponds to early work (Barron, 2001; Riddell et al., 2001) as well as more recent work (Johnson and Bagatell, 2020; Safta-Zecheria and Independent researcher, 2018) suggesting that people with intellectual disabilities by tradition have been infantilized and deprived adult social status and responsibilities.

Previous studies have provided information from relatives and staff on objective living conditions for people with intellectual disabilities. Both at baseline measurement (Umb-Carlsson and Sonnander 2005) and at follow up (Umb Carlsson, 2020) comparisons with the general population showed differences to the disadvantage of people with intellectual disabilities. It was suggested that living conditions were based on a societal view that people with intellectual disabilities had common needs rather than formed by individual preferences and needs. However, objective living conditions are not enough to describe QoL and a sense of wellbeing. Therefore, our study complements this previous work by focusing on people with intellectual disabilities themselves and their subjective experiences of living conditions.

Like most adults, study participants in our study had their own home, either in an ordinary dwelling or a group home. However, the findings indicate that their daily lives differ from what is considered a normal adult life. Several activities, usually conducted in the private home, were initiated by staff and held jointly in common areas (e.g., eating dinner on weekdays, watching TV). Although sharing meals is something that most people do with relatives and friends, it was not obvious for people with intellectual disabilities to influence with whom or when to share meals. This finding agrees with previous studies indicating that staff in housing control matters such as meal arrangements, including plans and preparations (Adolfsson et al., 2012; Adolfsson et al., 2010). These findings points at the dilemma of a group home as a private apartment at the same time as a workplace for staff.

Previous findings indicate that support can hinder living according to personal preferences (Sheth et al. 2019; Umb Carlsson and Adolfsson, 2018). In our study, study participants felt encouraged to make their own choices but within the framework set by staff and family. However, the choices of individuals residing in a group home often had to take count of the choices of other residents. This finding agrees with previous results (Adolfsson et al., 2010; Umb-Carlsson and Sonnander, 2005) indicating that aspects of institutionalization, where people with intellectual disabilities are treated as a homogenous group, still exist. In addition, it is argued that structural barriers with limited management and tutorial for staff may hinder changing established working methods, autonomy and society participation (Bigby and Wiesel, 2019; Chou et al., 2008; Fulford and Cobigo, 2018; Kozma et al., 2009). However, the picture in this study also indicates steps have been taken to strengthen adult responsibility. Perhaps the clearest example was that people in ordinary dwellings had the power to choose staff and manage their staff support. Still, one implication for the practice is to promote structure and skills for those living in a group home to influence their support and staff.

A key component of wellbeing is to be included in a social community with mutual interpersonal relationships with staff, family members and co-habitants (Gilmore and Cuskelly, 2014; Strnadová et al., 2018; Wilson et al., 2017). It is widely recognized that people with intellectual disabilities have fewer chosen friends than the general population (Umb Carlsson, 2020) and rarely associate with anyone outside the group they live with (Umb Carlsson and Adolfsson, 2018). Our study confirms this common belief. Study participants told about staff providing social support to increase interaction between co-residents. However, they did not mention anything about receiving skills and support to create and maintain friendship beyond co-residents, staff and family. An important implication for practice may be to develop support mechanisms to expand the social network of adults with intellectual disabilities. Wilson and colleagues (2017), for instance, showed that interventions such as forming a social group might expand the social life of people with intellectual disabilities and contribute to social belonging and community participation.

Although a positive interaction with co-habitants contributed to wellbeing, study participants emphasized the importance of the relations with staff and family. However, they distinguished between their relationship with staff and their family relationship. They felt confidence in the staff but they felt more linked to parents and other family members. Study participants had full trust in family members, a trust built on continuity and reciprocal emotional relations. If parents could not pay a visit or otherwise keep in touch regularly, they would ensure that other family members could. The importance of family relations has also been shown in previous research (Van Asselt-Goverts et al., 2015; Wilson et al., 2017) indicating that the relation may have a critical impact on establishment of a social network and community participation (cf. Wilson et al., 2017). Study participants who lacked contact with the family said that they missed it and, if possible, wanted support to establish such a vital connection (cf. Knox and Bigby, 2007). An implication for practice may be to encourage and stimulate existing family relations. For those who lack but want family ties, support in establishing informal relations is of vital importance.

The relationship between staff members and adults with intellectual disabilities is a dynamic process, which require a knowledge-based approach among staff. An implication for practice is to ensure that all staff have relevant education. Good treatment and quality of life may be different things to different people, depending on individual needs and preferences. Including the views of people with intellectual disabilities directly in everyday decisions would facilitate implementation of a personalized support for adults with intellectual disabilities.

### Limitations

Several limitations should be noted when interpreting the findings. A major limitation is that the small number of study participants residing in the same region may not represent general experiences of living conditions in other areas. Thus, the choice of study participants may have had an impact on our results. Although there was heterogeneity for several factors (e.g., socio-demographic variables, housing location, type of service and support in everyday life and overall life situation), the choice of study participants may limit transferability to other contexts. Nor did the number and choice of participants allow comparisons between groups.

Another limitation is the ambition to obtain self-reported experiences of living conditions. The desire to include first-hand information of individuals with intellectual disabilities is challenging because these people often have limited communicative skills. This lack of communicative skills was also observed in the study in which some study participants were more verbose than others and not used to contributing their feelings and thoughts about perceived living conditions. The presence of a support person and the desire to answer “correctly” are other factors that may have affected the study participants’ answers. In addition, the support persons’ preunderstanding may have influenced their interpretation when providing communicative support.

The interview questions and the UQoL2 were compiled in close cooperation with people with intellectual disabilities taking their views and preferences into account. Both authors have many years of work and research in the field. Thus the study likely captured key aspects that matter for those concerned. However, people with intellectual disabilities did not participate in data analysis and interpretation of the data. Together with various staff groups, they have been informed about the results at seminars and had the opportunity to comment on these.

## Conclusion

The study provides information on living conditions from people with intellectual disabilities’ perspective and complements previous research assessing the standpoint of staff and relatives (Umb Carlsson, 2020; Umb Carlsson and Sonnander, 2005). The findings show that the dependence on support in daily life infringes on the sense of adult social status and the possibilities of a life based on one’s own needs and preferences. A key component of wellbeing is to be included in a social community with staff, friends and family. Knowledge about factors that affect living conditions, one of the social determinants of health, has implications for public health in general and the development of society’s efforts for people who are in lifelong dependence on support and service from others.

## Appendix 1 (translated version)

1. What is your name?
2. Do you have any impairment?
3. Do you manage by yourself when being outdoors? In the nearby hood? At another place?

### Employment/work

4. Do you work? Where do you work?
5. Do you enjoy your job?
6. Do you feel safe at work?
7. Would you like to work more/less?
8. Do you have fun tasks?
9. Do you want to change job? What would you like to do?
10. Do you think your job is difficult enough?
11. Can you work at your own pace?
12. Can you stay at home when you are sick?
13. Is there something you want to change in your job? What?

### Finances

14. From where do you get your money/your income?
15. Do you get paid for your job? How much?
16. Do you have enough money to buy clothes? Have you been forced to refrain from buying clothes because you could not afford them?
17. Do you have enough money to meet relatives and friends? Have you been unable to meet relatives and friends because you could not afford it?
18. Do you have enough money to do what you want? Have you had to refrain from participating in a leisure activity because you could not afford it/ had no money?
19. Have you in the past year failed to pay the rent because you could not afford it?
20. Do you have a computer? Can you afford to have a computer?
21. Do you have Internet? Can you afford to have Internet?
22. Do you handle your money yourself? Would you like to handle your money yourself?

### Housing

23. Do you live in your own apartment? Do you live in a group home?
24. Do you co-habit? With whom?
25. With what do you need help? Who provides the help?
26. Do you have your own kitchen?
27. Do you have your own toilet?
28. Who cleans?
29. Who washes?
30. Who buys the food?
31. Can you choose which staff will help you?
32. Who have chosen your furniture?
33. Do you think that your apartment is too small/moderate/to big?
34. Do you have a key to your apartment?
35. Can you get out yourself if there is a fire? If not, is there anyone who can help you?
36. Do you have to ask staff if you want to go out in the evening?
37. Do you feel comfortable at your home?
38. Are you allowed to have guests?
39. Can you move if you want to?

### Personal safety

40. Do you feel safe at home?
41. Do you feel safe at work?
42. Have you been exposed for violence? At home, at work, outdoors?

### Leisure activities

43. What do you do when you are not working?
44. Have you travelled on a holiday trip during the past year?
45. How long were you away then?

### Family and social relations

46. Are your parents alive?
47. Do you have siblings in life?
48. Do you have children? How many? Do they live at your place?
49. Do you have a trustee?
50. Do you have a contact person?
51. Do you have a companion person?
52. Do you use Facebook, Instagram, Snapchat or similar to have contact with friends? How often?
53. How often do you meet your mother?
54. How often do you talk to her on the phone or on Skype?
55. How often do you meet your father?
56. How often do you talk to him on the phone or on Skype?
57. How often do you meet your siblings?
58. How often do you talk to them on the phone or on Skype?
59. Do you have a friend you can trust and talk to about anything?
60. Do you meet your neighbors?
61. Do you sometimes help your neighbors? Do they help you?
62. Are you member of any association? If yes, which?
63. Have you participated in any activity that the association has arranged?
64. Did you vote in the last election?

### Other

65. Other. Is there anything more you want to tell?
